# How have governments supported citizens stranded abroad due to COVID-19 travel restrictions? A comparative analysis of the financial and health support in eleven countries

**DOI:** 10.1186/s12879-022-07155-2

**Published:** 2022-02-20

**Authors:** Pippa McDermid, Adam Craig, Meru Sheel, Holly Seale

**Affiliations:** 1 School of Population Health, Faculty of Medicine, University of New South Wales, Level 2, Samuels Building, Sydney, NSW 2052 Australia; 2 Sydney School of Public Health, Faculty of Health and Medicine, the University of Sydney, Sydney, NSW Australia

**Keywords:** COVID-19, Pandemic, Communication, Travel

## Abstract

**Background:**

In response to the continuing threat of importing novel coronavirus disease (COVID-19), many countries have implemented some form of border restriction. A repercussion of these restrictions has been that some travellers have found themselves stranded abroad unable to return to their country of residence, and in need for government support. Our analysis explores the COVID-19-related information and support options provided by 11 countries to their citizens stranded overseas due to travel restrictions. We also examined the quality (i.e., readability, accessibility, and useability) of the information that was available from selected governments’ web-based resources.

**Methods:**

Between June 18 to June 30, 2021, COVID-19-related webpages from 11 countries (Australia, New Zealand, Fiji, Canada, United States of America (USA), United Kingdom (UK), France, Spain, Japan, Singapore, and Thailand) were reviewed and content relating to information and support for citizens stuck overseas analysed. Government assistance-related data from each webpage was extracted and coded for the following themes: travel arrangements, health and wellbeing, finance and accommodation, information needs, and sources. Readability was examined using the Simplified Measure of Gobbledygook (SMOG) and the Flesch Kincaid readability tests; content ‘accessibility’ was measured using the Web Content Accessibility Guidelines (WCAG) Version 2.1; and content ‘usability’ assessed using the usability heuristics for website design tool.

**Results:**

Ninety-eight webpages from 34 websites were evaluated. No country assessed covered all themes analysed. Most provided information and some level of support regarding repatriation options; border control and re-entry measures; medical assistance; and traveller registration. Only three countries provided information or support for emergency housing while abroad, and six provided some form of mental health support for their citizens. Our analysis of the quality of COVID-19-related information available on a subset of four countries’ websites found poor readability and multiple accessibility and usability issues.

**Conclusion:**

This study uniquely analyses government support for citizens stuck abroad during the COVID-19 pandemic. With large variance in the information and services available across the countries analysed, our results highlight gaps, inconsistencies, and potential inequities in support available, and raise issues pertinent to the quality, accessibility, and usability of information. This study will assist policymakers plan and communicate comprehensive support packages for citizens stuck abroad due to the COVID-19 situation and design future efforts to prepare for global public health emergencies.

**Supplementary Information:**

The online version contains supplementary material available at 10.1186/s12879-022-07155-2.

## Background

In response to the threats posed by the COVID-19 pandemic, countries around the world have imposed some level of international travel restriction for returning travellers [1–3]. Border restriction (or closure) was one of the earlier and most common non-pharmaceutical interventions implemented, with countries—including Australia, Italy, the Pacific Island countries and territories, and the USA—imposing some form of travel restrictions as early as February 2020 [1, 4]. Restrictions included suspending (or limiting) the number of international flights, suspending visa free travel as well as the use of enhanced quarantine and screening measures, and the issuing of recommendations to avoid non-essential travel [1–3, 5]. These restrictions were implemented to: (i) prevent importation of COVID-19 cases, including those with emergent strains of the virus; (ii) reduce the global spread of COVID-19 cases; and (iii) manage demand for border screening and quarantine services [1, 2, 6]. As an outcome, air travel reduced by nearly 70% in May 2020 compared to the previous year [3, 7], making it difficult for some citizens who were outside their home country to return.

Due to the decisions of governments to impose these restrictions and the subsequent challenges in accessing travel or repatriation options, many people, 16 months into the pandemic, have found themselves stranded abroad. This has included those abroad for tourism, business, family, education purposes, among others. While the number of stranded citizens will vary between countries, for Australia it was suggested that over 36,000 citizens were stranded overseas and required assistance to returning home in April 2021 [8]. This includes 500 people who were considered vulnerable [8]. However, this number probably an underestimate, as it only captures those who attempted to return home of their own accord [8]. In 2020, within 6-months of the COVID-19 pandemic, it was estimated that India had the largest number of repatriated citizens (more than 250,000), followed by the Philippines and USA [9]. Some countries opted not to repatriate their citizens because of numerous reasons including inadequate domestic quarantine capacity, and as of April 2021 many countries have either stopped repatriation completely, or offered limited availability impacting on the number of citizens stranded abroad [10]. While the evacuation and repatriation of citizens is most critical, Hugelius [11] and others have highlighted the important role national governments play in ensuring medical care, psychological, and financial support for their citizens abroad [11, 12].

To date, literature examining support made available to citizens stranded abroad have focused on international emergencies, including Ebola, civil unrest in the Middle East and natural disasters in New Zealand and Japan [11, 13–17]. Other works have focussed more broadly on general diaspora policies, as well as the recommendations around social protection and consular services for citizens abroad [15, 18–21]. To our knowledge, our research is the first to explore the variations in government support and services available to citizens stranded abroad during the COVID-19 pandemic. This study aims to compare the availability of information and support made available by 11 countries to help their citizens stranded abroad due to COVID-19-related travel restrictions. Further, the study will examine the quality of COVID-19 assistance-related information provided by a sub-sample of the countries.

## Methods

### Country selection

Eleven countries (Australia, New Zealand, Fiji, Canada, USA, UK, France, Spain, Japan, Singapore, and Thailand) were included in this study. Countries were selected based on the availability of English language government and consular websites. Other factors that were considered included countries that quickly implemented total border closures and restrictions (Australia, New Zealand, and Spain) compared to other countries that had a delayed response (UK and Japan) [22]. Additionally, population and geographical differences were also considered, with the USA bordering multiple countries with the largest populations in each respective continent, compared to the island nations of Singapore, Fiji, UK, Australia and New Zealand with drastically different case number and mortality rates. Finally, an important factor was the inclusion of low, middle, and high-income countries.

### Data collection and document analysis

Data were collected between June 18 to June 30, 2021, from publicly available government websites. These included central government, Ministry of Health, consular and embassy websites.

To identify relevant websites, we conducted a manual search through Google using the country name along with the following terms: repatriation flights, border control and re-entry information, mental health support, medical assistance, government financial assistance, emergency housing, registration information and any citizen forums available. Once the website (collection of multiple webpages) was located, we then systematically searched all of the webpages (a single document on the web using a unique URL) with the previously mentioned themes to identify potentially missed pages or publicly available downloadable materials. We then reviewed and extracted data from the websites onto an excel spreadsheet and depending on their content, coded them to one or more of the following, pre-determined focus areas based on traveler sentiment in the media: Travel Arrangements, Health and Wellbeing, Finances and Accommodation, Information needs and sources. We then conducted a document analysis based on the excel spreadsheet as a method for evaluating the similarities and differences between services and support available to citizens abroad of 11 countries.

### Information quality data analysis

Four countries (Australia, Canada, France, UK), were selected for further analyses, based on them having clearly dedicated COVID-19 web pages specifically for citizens abroad located in a single website. With France having a total of four pages dedicated to COVID-19 for citizens abroad at the time of writing, for consistency, four COVID-19 dedicated webpages were randomly chosen from a search result set for ‘citizens abroad’ from each of the government websites of Australia, Canada, and the UK. In total16 webpages were assessed for readability, accessibility, and usability.

For this study, readability was defined as being the measure of reading difficulty of a given text, usability heuristics were defined as a measure of the general principles used to analyse webpage design to ensure usability, and accessibility of pages was assessed as a requirement for websites to be usable for those with disabilities and for those using other devices other than computers, including phones and tablets [12, 23–26]. Three validated tools, all previously utilised in assessing COVID-19 communications, were adopted for this present study [12, 27].

The Simplified Measure of Gobbledygook (SMOG) and the Flesch Kincaid readability tests were both used to test the information sourced from the dedicated webpages per country into the readability calculator (http://www.readabilityformulas.com/free-readability-formula-tests.php) [25, 28]. The score outlined by the SMOG test estimates the grade level needed to understand the text, with a score of 9 representing a 9th grade reading level. The Flesch Kincaid readability score however, represents the level of reading ease, with scores of 0 to 30 showing information that is ‘very difficult to read’, 30 to 50 being ‘difficult to read’, 50 to 60 as ‘fairly difficult to read’, 60 to 70 as ‘standard/average’, 70 to 80 as ‘fairly easy to read’, 80 to 90 as ‘easy to read’ and finally, 90 to 100 as ‘very easy to read’[25, 28]. Scores of both reading age given by the SMOG and reading level from Flesch Kincaid readability test were averaged by country to give an overall readability score and categorisation of reading ease, with research suggesting that year 8 in Australia and grade 6 in the USA maintains the most appropriate reading age for information comprehension across the general population [25, 27, 28].

The Web Content Accessibility Guidelines (WCAG) Version 2.1, an internationally accepted guideline for website accessibility compliance, was used for analysing accessibility for four webpages per country assessed (n = 16) [24]. The Wave tool was used, developed by WebAIM, to check accessibility compliance against WCAG 2.1 and produced a report per page which detailed the violations to the guidelines [24, 29]. Finally, usability was assessed based on two usability heuristics for website design, including assessing the visibility of system status of the webpages, represented by information being provided on the webpage on when it was last updated or published [26]. The match between system and the real world was also assessed by the availability of additional languages other than English [26].

## Results

We analysed and evaluated data from 11 countries and found that no country assessed covered all themes analysed. Most, however, provided some level of support regarding repatriation options; border control and re-entry measures; medical assistance; and traveller registration. The results presented here focus on publicly available information regarding support and services for citizens abroad that were developed by federal governments and local consulates of the 11 countries selected. (See Additional file 1: for a full breakdown of government information sources accessed).

### Information available on travel arrangements and border control measures

#### Repatriation flights

As of June 30, 2021, repatriation flights appeared to be still operating for citizens wishing to return to Australia, Thailand, Fiji, and the USA. However, there were no further details found outlining the number and timing of repatriation flights for any of the countries. Although the French Government offered repatriation flights for citizens abroad in early 2020, it was unclear through online government sources whether they had continued. Government sources for Canada, Spain, the UK and Japan outlined that although evacuations and repatriation flights had operated during the ongoing COVID-19 situation, they were not being continued at the time the webpages were reviewed in June 2021. We found no evidence that reparation flights had or will occur for Singapore and New Zealand citizens.

A press release from the Japanese Foreign Minister Motegi, speaking at the 2nd Tokyo Global Dialogue on ‘Japan’s foreign policy in a post COVID-19 world’ in February 2021, stated that one of his most vital responsibilities was in protecting Japanese citizens abroad, with repatriation flights beginning in January 2020 and returning approximately 12,000 Japanese citizens from around 101 countries [30]. Although reparation and evacuation flights remained available, if necessary, it was reported that as of November 2020 there were no Japanese citizens stranded abroad. Likewise, the Ministry of Foreign affairs for Spain, in collaboration with the European Union and Cooperation in a press release state, that repatriation and assistance is complete with the return of 25,000 Spaniards from around the world after multiple facilitated flight operations [31].

#### Border control and re-entry information

International border restrictions and information provided through government websites varied between the 11 countries examined (see Additional file 2. for a full breakdown of border restrictions, advice on quarantine, screening and who could enter). As of June 2021 (when our analysis was conducted), 5/11 countries had strict border closures in place, allowing only citizens or residents entry, enforceable testing, and quarantine requirements. Of these five, only Australia and New Zealand limited the number of passengers that could enter their respective home country, with New Zealand requirements based solely on availabilities of booking for hotel quarantine. The remaining six countries varied in the level of border restriction in terms of who can enter and for what reason, with evidence suggesting that some travel for tourism had resumed.

### Support and information available on health and wellbeing

#### Mental health

Government pages dedicated to COVID-19 support for citizens of Australia, Canada, and the UK all included detailed resources and referrals to mental health support services while abroad. All three government webpages provided emergency numbers, services and contacts that were available 24/7.

Additionally, each of the three government pages had links to detailed advice and tips on looking after mental health and wellbeing during COVID-19, with both the Australian and UK governments providing recommended phone applications and tools listed as additional mental health support options. Each link to further resources and tools were visible and up to date. The Government of Canada webpage indicated a recent update of June 2021, while the UK webpage had not been updated since 2020, and the Australian webpage had no date listed.

Mental health advice for New Zealand citizens was available on the government webpage ‘Unite against COVID-19’ and gave advice on how to maintain mental wellbeing in times of stress, where to go if you need professional help and free smart phone applications, online resources, and toolkits. Notably, this advice was not specific to citizens abroad but citizens and residents of New Zealand as a whole, with page updates ranging from June 2020 to January 2021. For USA citizens abroad, the Centers for Disease Control and Prevention (CDC) website had a page on ‘Mental Health and Travel', with no emergency contact details besides recommendations to seek assistance from your local USA Embassy during your travel. A search of support available on the USA Embassy & Consulates in Australia, resulted in suggested local service provider hotlines, with phone numbers for Lifeline and the Mental Health Help Line being the only provided mental health support service, with the webpage at the time specifying that services listed are not endorsed by the USA Government. No separate advice or resources were provided on mental health besides the contact numbers.

Government sources in France, Spain, Fiji, Japan, Singapore and Thailand did not appear to include any mention of mental health support or guidance for citizens abroad.

#### Medical assistance

Emergency assistance information, including consular contact numbers were available for citizens of all 11 countries, found either in an emergency abroad page or in the contact information on each consulate and government page. Government pages offering advice on medical and emergency assistance abroad, was only available on ten of the 11 countries’ webpages, with some being more detailed than others. The governments of Australia, Canada, New Zealand, and the UK gave details of a 24-h consular emergency line for citizens abroad on COVID-19 specific pages.

Only the governments of Canada and the UK had pages dedicated to health and safety during COVID-19 whilst abroad. These pages included information on vaccinations, symptoms, and other COVID-19 related health information. The Japanese and Singapore government pages including Ministry of Foreign Affairs and local consulates in Australia did not have clear sections within the main page or after a search that indicate advice surrounding seeking medical assistance abroad.

### Support and information available on finances and accommodation

#### Government financial assistance

In terms of financial assistance for citizens abroad, 5/11 countries’ government webpages included information on available loans or grants with varying rules and application processes, with the French government standing out with having the most available cash benefit schemes for citizens abroad, both prior to and since COVID-19 (see Table 1).

**Table 1:** Comparison of financial assistance offered to citizens abroad in six countries as of June 2021

Country	Financial assistance	Finance Type (Loan/Grant)	Who can apply?	Repayments
Australia	1. Loan that covers living costs until a flight becomes available 2. If currently receiving Pension payments, or for children under 18, a grant is available for both emergency living expenses and an airline ticket 3. Loan that covers part of the flight ticket costs	Loans and Grants	• Those who have been unable to return to Australia due to travel restrictions who are feeling financial distress • Those who want to return rapidly for medical reasons (with proof of medical condition provided) • Australian citizen or Australian permanent resident whose application is completed by an Australian citizen • Must provide evidence to confirm that financial support was not available elsewhere including Bank/credit card statements, travel agent, employer (proof of termination or lack of leave payments), local social security services, a pension from overseas, insurance company (letter confirming they will not pay), and family and friends	All loans must be repaid within 6 months of arrival, or a payment plan being initiated within 6 months
Canada	1. The COVID-19 Emergency Loan Program of up to $5000	Loan	• For Canadian citizens or permanent residence of Canada who have been unable to return or find suitable accommodation whilst awaiting return • Must prove that other sources of financial assistance have been attempted, including banks and credit cards, and possible transfers from family and friends	Must be repaid within 180 days from the date on the invoice
France	1. Government funded Charities—Local Associations of assistance and solidarity (Organismes locaux d’entraide et de solidarité) 2. Fixed-term social allowance (Allocation à durée déterminée, ADD) 3. Child-specific scheme (Secours mensuels spécifiques enfants)—one-time payment 4. Occasional aids (Secours occasionnels)—covers expenses to citizens facing exceptional circumstances 5. COVID-19 Social Assistance System for those affected by economic crisis due to COVID-19 abroad (SOS)—up to 259 Euros per month	Financial Aid and Cash benefits	• For French citizens abroad facing financial hardships • To claim SOS, French citizens must be abroad and registered under ‘French people living outside France’ and can prove a recent loss of income or if they are in an emergency	Does not appear to need to be repaid
UK	1. FCDO Emergency loan for repatriation to the UK—usually to cover a ticket	Loan	• Must prove that other sources of financial assistance have been attempted, including from travel insurance companies, banks and credit cards, charities (e.g. Turn2Us), fundraising, employers and possible transfers from family and friends • Only as a last resort in case of destitution • Must be a British citizen of have lived in the UK for at least 5 years normally	All loans must be repaid within 6 months
USA	1. Limited medical assistance loans in case of an emergency 2. Consular officer can help apply for loans to cover emergency accommodation and other essential expenses	Loan	• For U.S citizens and qualified dependents • Must have attempted at the first instance to access financial support from friends and family, banks or employer	Must be repaid

*COVID-19* novel coronavirus disease; *FCDO* Foreign, Commonwealth & Development Office; *UK* United Kingdom; *USA* Unites States of America

Comparatively, New Zealand did not offer financial assistance for citizens overseas, with a government page stating that “New Zealanders overseas have no right or claim to financial help from the New Zealand Government” [32]. The exception to this was the continuation of social development payments. Information on what to do if New Zealand citizens find themselves suffering financial distress overseas was available, and included major bank contact numbers and advising to check with employer/insurance company. Similarly, both Fiji and Singapore did not provide any loans, but citizens abroad could request the government to seek financial support from friends and family on the citizens behalf if required. It is notable however, that Fiji would provide a loan for emergency replacement of travel documents.

Citizens of Spain were advised to contact the local consular office to seek further assistance in exceptional circumstances. It is unclear whether the governments of Japan or Thailand provided emergency financial assistance for nationals overseas. However, the Japanese Ministry of Foreign Affairs had created a fund for projects aimed at supporting Japanese nationals abroad whose lives have been affected by COVID-19, called the Grant Program to Strengthen Livelihood and Business Foundations for Japanese Nationals Overseas and People of Japanese Descent (Nikkei).

#### Emergency housing

Only the governments of Spain and France appeared to have developed a solution to emergency housing needs for citizens in need stuck abroad. Both countries had similar design initiative, with Spanish or French nationals either hosting citizens in need or requesting emergency accommodation on the respective platforms. However, the link for the service offered to French citizens via www.sosuntoit.fr could not be reached using multiple web browsers. Conversely, although no official temporary housing solution was available, the British government provided advice for citizens without accommodation and encouraged speaking to the local British consulate or high commission for further advice.

### Support and information available for citizen/traveller registration

Overall, 9/11 countries examined provided a form of registration focused on supporting citizens abroad, whether through information sharing, alerts, or the ability for local consulate support (see Table 2).

**Table 2:** Comparison of government registration and what that provides citizens abroad in 11 countries as of June 2021

Country	Registration name	What it provides
Australia	Department of Foreign Affairs and Trade portal (DFAT)	• Provides a portal for Australian citizens who wish to return to register details to assist the Australian government to better understand who is wanting to come home for planning purposes to prioritise vulnerable citizens
Canada	Canadians Abroad and the Travel Smart App	• This registration allows the Canadian Government to update citizens in case of emergencies both at home and in the country, they are currently residing in • Important information before or during a crisis is also provided, including instructions, and changes or updates to the travel advice for the country listed on the registration form • The Travel Smart app will provide travel advice for the country citizens are either residing or travelling in
France	Registry of French residing abroad	• An alternative way to complete administration formalities • Access to services available to French citizens abroad • Consular information • A way to contact your family in case of an emergency
Japan	Overseas Travel Registration (Tabi-Regi)	• Labelled one of the most important initiatives to ensure the safety of Japanese citizens abroad, this registration provides citizens with frequently updated travel safety advice and information • Ability for local consulate to confirm safety during a crisis
New Zealand	SafeTravel registration	• Provides citizens with important updates and information on the situation in countries worldwide • Ability for local consulate to confirm safety during a crisis
Singapore	Ministry of Foreign Affairs e-register	• Will help the local Singapore embassy to assist citizens in case of an emergency • Provide disseminating information on important embassy services • Ability for local consulate to confirm safety during a crisis
Spain	Local Consular Registration	• Document renewal • Citizens can request consular assistance through this registration • Participate in elections • Ability for local consulate to confirm safety during a crisis • Can connect families to citizens through the consulate via this registration in a crisis
Thailand	Local Consular Registration	• Information collected to be used for the local Thai Embassies to assist citizens in case of an emergency • Ability for local consulate to confirm safety during a crisis
USA	Smart Traveler Enrollment Program (STEP)	• Information provided on conditions in the multiple country abroad U.S citizens are located in. Conditions can include warnings, areas of concern, where and how to seek assistance for example • Alerts to leave areas in severe situations, including local consulates identifying transportation options out of the crisis area

*USA* Unites States of America

Registration to a government run portal or application did not appear to be available to citizens of Fiji or UK. However, for UK citizens abroad, government advice suggested signing up to the Foreign, Commonwealth and Development Office email alerts. Unique to Singapore was the government supported Singapore Global Network (SGN), created to increase engagement, support, and connection with Singaporeans abroad. Within the webpage, SGN provided detailed information on COVID-19, both for citizens wanting to return to Singapore and the steps needed, to those wanting to remain based overseas. Signing up to SGN provided citizens with frequent vital updates.

### Information quality analysis on readability, accessibility, and usability of resources

#### Readability

Of the 16 pages assessed for readability, the overall mean SMOG score was 9.71 (range 7.1–12), representing a reading grade of the tenth grade. Results of the Flesch Kincaid reading ease score show an average of 51.95 (39–65.6), indicating that the pages were on average ‘fairly difficult to read’, representing a reading grade of between tenth-twelfth grade (see Table 3). The French government resources (n = 4) had the best readability scores, showing a mean SMOG score of 8.45 (range 7.1–9.7) or eighth grade and a Flesch Reading Ease mean score of 45.25 (range 39–51.6) indicating that the web pages were difficult to read, and a Flesch Reading Ease mean score of 57.2 (range 49–65.6) indicating that the web pages were reasonably difficult to read. In comparison, the Australian Government’s resources represented the worst readability scores, with a mean SMOG score of 10.5 or eleventh grade, and a Flesch Reading Ease mean score of 45.25 (range 39–51.6) indicating that the web pages were difficult to read.

**Table 3:** An overview of the range and mean SMOG and Flesch Readability Ease scores for four countries

Country	Range and mean	SMOG	Flesch Reading Ease score	Flesch Readability ease
Australian Government *(n* = *4)*	Range: Mean:	9–12 10.5 (Grade 11)	39–51.6 45.25	Fairly difficult to read -difficult to read Difficult to read
Canadian Government *(n* = *4)*	Range: Mean:	7.4–12 10.33(Grade 10)	40.1–59.5 49.38	Standard/average—Difficult to read Difficult to read
UK Government *(n* = *4)*	Range: Mean:	8.3–11.2 9.58(Grade 10)	52–63 55.95	Standard/average—Fairly difficult to read Fairly difficult to read
French Government *(n* = *4)*	Range: Mean:	7.1–9.7 8.45(Grade 8)	49–65.6 57.2	Standard/average—Difficult to read Fairly difficult to read
All webpages *(n* = *16)*	Range: Mean:	7.1–12 9.71(Grade 10)	39–65.6 51.95	Standard/average—Difficult to read Fairly difficult to read (10th–12th Grade)

*SMOG* Simple Measure of Gobbledygook; *UK* United Kingdom

#### Accessibility and usability

Analyses of the 16 webpages of the four countries assessed, detected 49 violations across 8 guidelines (see Table 4 for website violations and guideline definitions). Overall, the number of errors, as seen in Table 4, remain minimal with 50% of countries having more than the observed median of 2 violations (range 1–28). The most common violation detected was a ‘contrast error’ (recommended website contrast minimum) accounting for 65% of the accessibility breaches. Usability evaluation showed that only 50% of the government webpages supplied visibility of system status (i.e., update information), although it is notable that the French government provided publication date. Only 50% of countries provided evidence of an additional language other than English. The Australian government pages assessed had neither an additional language nor update information available within the webpage design (see Table 5).

**Table 4:** Accessibility WCAG 2.1 Guidelines violated by evaluated government websites

WCAG Guideline Reference	Number of Violations per country			
	Australian Government	Canadian Government	UK Government	French Government
*Empty Headings* [1.3.1 Info and Relationships (Level A; 2.4.1 Bypass Blocks (Level A); 2.4.6 Headings and Labels (Level AA)]	1			
*Missing Alternative Text* [1.1.1 Non-text Content (Level A)]		1	1	
*Broken Skip Link* [2.1.1 Keyboard (Level A); 2.4.1 Bypass Blocks (Level A)]		2		
*Empty Button*** [**1.1.1 Non-text Content (Level A); 2.4.4 Link Purpose (In Context) (Level A)]				12
*Contrast Error* [1.4.3 Contrast (Minimum) (Level AA)]		4		28
Total Violations	1	7	1	40

*UK* United Kingdom; Web Content Accessibility Guidelines 2.1; Level A, Minimal compliance requirement; Level AA, Acceptable compliance recquirement

**Table 5:** Overview of WAVE detected WACG 2.1 violations and usability heuristics for four countries

Country	Errors	Alerts	Contrast errors	Update information	Languages other than English	Sources
Australian Government *(n* = *4)* Total	0 1 0 0 1	13 15 15 15 58	0 0 0 0 0			1. https://www.smartraveller.gov.au/COVID-19/trying-get-home 2. https://www.smartraveller.gov.au/COVID-19/trying-get-home/facilitated-flights 3. https://www.smartraveller.gov.au/COVID-19/COVID-19-overseas-financial-assistance 4. https://www.smartraveller.gov.au/covid-19/covid-19/covid-19-re-entry-and-quarantine-measures
Canadian Government *(n* = *4)* Total	1 0 1 1 3	3 3 3 3 12	1 1 1 1 4	✓ ✓ ✓ ✓	✓ ✓ ✓ ✓	1. https://travel.gc.ca/assistance/emergency-info/financial-assistance/covid-19-financial-help 2. https://www.canada.ca/en/public-health/services/diseases/2019-novel-coronavirus-infection/mental-health.html 3. https://travel.gc.ca/travelling/registration 4. https://travel.gc.ca/travelling/health-safety/covid-19-security#help
UK Government *(n* = *4)* Total	0 0 1 0 1	5 2 2 2 9	0 0 0 0 0	✓ ✓ ✓ ✓		1. https://www.gov.uk/guidance/coronavirus-covid-19-staying-where-you-are-if-you-cannot-return-to-the-uk 2. https://www.gov.uk/guidance/wellbeing-and-mental-health-during-the-coronavirus-covid-19-pandemic 3. https://www.gov.uk/government/publications/financial-assistance-abroad/financial-assistance-abroad 4. https://www.gov.uk/guidance/healthcare-support-for-when-you-are-unable-to-return-to-the-uk-during-coronavirus-covid-19
French Government *(n* = *4)* Total	3 3 3 3 12	12 9 10 15 46	7 7 7 7 28		✓ ✓ ✓ ✓	1. https://uk.ambafrance.org/COVID-19-rules-for-travel-to-France-and-the-UK 2. https://uk.ambafrance.org/NHS-COVID-Pass-now-accepted-in-France 3. https://uk.ambafrance.org/Strategy-for-reopening-of-borders-from-9-June-onwards 4. https://uk.ambafrance.org/COVID-19-Adaptation-of-consular-services-from-2-June-2020-onwards
All webpages *(n* = *16)*	17	125	32			

*UK* United Kingdom; *WACG 2.1* Web Content Accessibility Guidelines 2.1

## Discussion

In this study, we examined available information on government support and assistance available to citizens abroad of 11 countries during the COVID-19 pandemic. We also analysed the quality of information provided by governments, examining the readability, usability, and accessibility for selected webpages on four countries’ COVID-19-related websites. From our review of government webpages, the most commonly reported support types were repatriation, information on border control measures, medical assistance, and registration of citizen details, with considerable variance. We also found that most countries did not have emergency housing support available during COVID-19, despite having safe shelter a fundamental aspect in crisis support [14].

Providing emergency shelter remains a vital humanitarian need within disaster plans across the world, especially during natural disasters. However, this accommodation is generally short term and focused on domestic disasters, including earthquakes and floods [33–35]. Recent evidence in Australia has shown that temporary migrants were already unable to access the national healthcare and welfare benefits during the beginning of the pandemic, and were reported as being at higher risk for potential homelessness, and housing and financial insecurity during COVID-19 [36, 37]. This issue has also been documented in Europe, where migrants were overly represented in the amount of rough sleepers around larger European cities [38]. It is essential that all governments consider the needs of those unable to access health and welfare support, by providing alternative emergency housing solutions, like those by the French and Spanish governments, as a major facet of both pandemic plans and emergency response management. Governments could consider expanding funding for housing and homelessness support and services, including rent relief eligibility and access for non-citizens.

Previous research has highlighted the psychological impacts of international emergencies and major events, including COVID-19, on not only the survivors but the respondents and those not directly affected [39–43]. More recently, a study looking into the mental health of Saudi citizens living abroad, found that two-thirds experienced symptoms of anxiety and depression [44]. Given this finding, it was reassuring to see the focus on mental health and mental wellbeing on the government websites of Australia, Canada, and the UK, especially the detailed advice and inclusion of services, contact information and applications available internationally. However, there were variations in the information and services provided, with the USA government for example, providing only minimal information. Whereas, the Canadian Government had created a ‘Wellness Together Canada’ platform, dedicated to supporting mental health and substance use during COVID-19 for Canadians both abroad and in Canada. The service is free to use and available 24/7, and provides the tools and resources to help tackle the increased demands in mental health services. Based on this review we recommend that all governments consider the inclusion of detailed mental health support and advice for citizens abroad, as well as the application of similar online services and mental health resources as shown by Canada.

Our results showed large variation in the availability and types of financial assistance available to citizens abroad, with only 5/11 having assistance in some form of either cash benefits, loans, grants, or financial aid. Some of the financial assistance offered reflects those in previous reports [14, 18, 19]. However, others, like the COVID-19 emergency loan program in Canada and the COVID-19 Social Assistance System in France were both new additions to consular support during COVID-19. It was outside the scope of our study to assess the ease in which citizens abroad could apply for these financing options, nevertheless, most of the countries require proof that alternative sources of financial assistance were sought prior to applying, which could prove to be difficult. Further research should be conducted to assess whether citizens abroad requiring financial assistance were aware of these options and were able to access it.

In her research, Melissen [13] argues that one of biggest challenges to aiding citizens abroad is not the type of help provided, but the communication and access of this support. Our results suggest an overall poor readability score across the 16 web pages assessed and that content was pitched well above the recommended readability level of 6^th^ to 8^th^ grade targeting the general population.

All webpages assessed violated at least one accessibility design feature, vital for those with disabilities, and those not able to access these webpages on a computer, whether due to internet access or only having a phone to view. Furthermore, when assessing for usability, only webpages managed by the Governments of Canada and France provided evidence of both languages other than English and update dates.

This implies that information may not always be reaching or being understood by the population intended, similar to the literature looking into the readability of documents and health information for public consumption [23, 27, 45]. Moreover, as the information assessed in this paper was accessed through a computer, certain issues regarding digital inequalities are raised [46]. Do citizens abroad have access to working networks or the devices needed, or similarly do they have the skills to navigate these spaces to access and understand all the information provided, especially across the multiple websites as we found?

We therefore recommend that governments utilise the multiple free and easy to use applications and tools in readability, usability an accessibility to ensure the information they are communicating can be understood by all. Essential to this is ensuring the visibility of publication and recent update information, as this, along with having access to information in multiple languages, could help build trust and confidence in the information provided.

Possible explanations for the differences in support and services available across the countries analysed are the complications associated with COVID-19 affecting each country domestically, with some countries having strict border controls, coupled with wide diaspora of population [47, 48].

This study has several limitations. First, our data collection focused on information available on government websites only, and it is possible that information was missed if it was distributed through alternative channels including social media or through traveller registration apps [49–51]. Second, the SMOG and Flesch Kincaid reading ease tools used in this study were not specifically designed to assess online information as has been discussed in other research, however they are the most frequently used tools in assessments of online information readability due to a lack of validated alternatives [12, 23, 27, 49]. Furthermore, with only four webpages selected per country for information quality analysis, there is a possibility that the readability, usability, and accessibility scores would be lower or higher depending on the webpages. Third, as the data was collected over a short period, between June 18 to June 30, 2021, not during the first year of pandemic, it is possible that the services and information available may be different to what was available in 2020 and may have changed since June 2021, due to the evolving nature of pandemic information and the pages sourced may have changed or no longer be accessible. Last, our results focused on webpages available in English and in doing so may have missed services and support available in only the native language of the country assessed. Despite these limitations, this study provides both novel and valuable insights on the needs and gaps of support and services available to citizens abroad during a crisis.

## Conclusions

The findings have shown the similarities and differences across 11 countries’ government support available and has found no evidence that any country assessed had complete support and information available across the themes recognised as of June 2021. This study offers information that will assist foreign ministries, communicators, and policy makers to respond to the support and information needs of citizens abroad during the COVID-19 pandemic, and unable to leave travel due to restrictions. The insights generated also highlight issues that ought to be considered as part of emergency preparedness planning for future public health events of international health concern. Further research is needed to ascertain whether these services were accessed by citizens abroad, what impact they had and whether there is a need for other support not identified in this study.

## Supplementary Information

**Additional file 1.** Government information sources accessed between June 18 to June 30, 2021.

**Additional file 2.** Comparison of Border control and re-entry information provided to citizens abroad of 11 countries as of June, 2021.

## Data Availability

All data supporting this study is provided as supplementary information accompanying this paper.
