# Supplementary material for: How have governments supported citizens stranded abroad due to COVID-19 travel restrictions? A comparative analysis of the financial and health support in eleven countries
Source: BMC Infect Dis. 2022 Feb 20;22:161. doi: 10.1186/s12879-022-07155-2 (PMC8858437; doi:10.1186/s12879-022-07155-2)
Supplement: Supplementary file 1 — Additional file 1. Government information sources accessed between June 18 to June 30, 2021. [file 12879_2022_7155_MOESM1_ESM.docx]

**Additional file 1.** Government information sources accessed between June 18 to June 30, 2021

| **Australia - Total Sources: 9** | | |
| --- | --- | --- |
| **Source** | **Webpage Title** | **Reference URL** |
| Australian Government – Department of Foreign Affairs and Trade (DFAT) | COVID-19: Trying to get home | https://www.smartraveller.gov.au/COVID-19/trying-get-home |
| DFAT | COVID-19: Facilitated Flights | https://www.smartraveller.gov.au/COVID-19/trying-get-home/facilitated-flights |
| DFAT | COVID-19: Registration | https://www.smartraveller.gov.au/COVID-19/trying-get-home/COVID-19-registration |
| DFAT | COVID-19: Overseas financial assistance | https://www.smartraveller.gov.au/COVID-19/COVID-19-overseas-financial-assistance |
| DFAT | COVID-19 Re-entry and quarantine measures | https://www.smartraveller.gov.au/covid-19/trying-get-home/covid-19-re-entry-and-quarantine-measures |
| DFAT | COVID-19: Staying Overseas | https://www.smartraveller.gov.au/COVID-19/staying-overseas |
| DFAT | COVID-19: Mental Health and Wellbeing | https://www.smartraveller.gov.au/COVID-19/mental-health-and-wellbeing |
| DFAT | COVID-19 Frequently asked questions | https://www.smartraveller.gov.au/COVID-19/COVID-19-frequently-asked-questions |
| DFAT | Medical Assistance Overseas | https://www.smartraveller.gov.au/while-youre-away/when-things-go-wrong/medical-assistance |
| **Canada - Total Sources: 11** | | |
| **Source** | **Webpage Title** | **Reference URL** |
| Government of Canada | COVID-19: Financial help if you are outside Canada | https://travel.gc.ca/assistance/emergency-info/financial-assistance/covid-19-financial-help |
| Government of Canada | Request emergency assistance | https://travel.gc.ca/assistance/emergency-assistance |
| Government of Canada | Sickness or injury | https://travel.gc.ca/assistance/emergency-info/sick-injured |
| Government of Canada | Canadian Consular Services Charter | https://travel.gc.ca/assistance/emergency-info/consular/canadian-consular-services-charter#assisting |
| Wellness Together Canada | Mental Health and Substance use support | https://wellnesstogether.ca/en-CA?lang=en-ca |
| Government of Canada | Global Affairs | https://www.canada.ca/en/global-affairs/news/2020/03/government-of-canada-to-provide-financial-assistance-to-canadians-abroad.html |
| Government of Canada | Use ArriveCan to enter Canada | https://www.canada.ca/en/public-health/services/diseases/coronavirus-disease-covid-19/arrivecan.html |
| Government of Canada | Travel Health notice | https://travel.gc.ca/travelling/health-safety/travel-health-notices/221 |
| Government of Canada | Registration of Canadians Abroad | https://travel.gc.ca/travelling/registration |
| Government of Canada | Find out if you can enter Canada | https://www.canada.ca/en/immigration-refugees-citizenship/services/coronavirus-covid19/travel-restrictions-exemptions.html |
| Government of Canada | COVID-19: Your safety and security outside Canada | https://travel.gc.ca/travelling/health-safety/covid-19-security |
| **Fiji - Total Sources: 5** | | |
| **Source** | **Webpage Title** | **Reference URL** |
| The Fijian Government | Media release – Families thank Fijian Government for repatriation | https://www.fiji.gov.fj/Media-Centre/News/FAMILIES-THANK-FIJIAN-GOVERNMENT-FOR-REPATRIATION |
| COVID-19 Risk Mitigation Taskforce | (PDF) Quarantine Charges for non-essential travel by Fijians | https://www.mcttt.gov.fj/wp-content/uploads/2021/03/CRMT-Press-Statement_New-Quarantine-Charges-for-returning-Fijians-English.pdf |
| The Fijian Government – Ministry of Foreign Affairs | Republic of Fiji’s Diplomatic Missions Abroad | http://www.foreignaffairs.gov.fj/missions-overseas/missions |
| Fijian Immigration Department | Getting Help Overseas | http://www.immigration.gov.fj/index.php/travel-options/fijians-travel-abroad/getting-help-overseas |
| Fijian Ministry of Health and Medical Services | (PDF) Fiji COVID-19 Border Control Measures | http://www.health.gov.fj/wp-content/uploads/2020/08/Fiji-COVID-19-Border-Control-Measures.pdf |
| **France - Total Sources: 10** | | |
| **Source** | **Webpage Title** | **Reference URL** |
| France Diplomacy | Press release | https://www.diplomatie.gouv.fr/en/coming-to-france/coronavirus-advice-for-foreign-nationals-in-france/coronavirus-statements/article/press-release-introduction-of-a-web-solution-to-help-french-citizens-in |
| Consulate General of France in the London | Sign up to the French citizens register | https://uk.ambafrance.org/Sign-up-to-the-French-citizens-register |
| French Government | Coronavirus (2019-nCoV) - Repatriation operations involving direct flights organized by France | https://www.gouvernement.fr/en/coronavirus-2019-ncov-repatriation-operations-involving-direct-flights-organized-by-france |
| Consulate General of France in the London | Relaxation of criteria for relief to help French nationals abroad | https://uk.ambafrance.org/Relaxation-of-criteria-for-relief-to-help-French-nationals-abroad |
| Consulate General of France in the London | Special COVID-19 assistance | https://uk.ambafrance.org/Aide-speciale-CoVid-19 |
| Consulate General of France in the London | COVID-19: rules for travel to France and the UK | https://uk.ambafrance.org/COVID-19-rules-for-travel-to-France-and-the-UK |
| Consulate General of France in the London | COVID-19: Strategy for reopening of borders from 9 June onwards | https://uk.ambafrance.org/Strategy-for-reopening-of-borders-from-9-June-onwards |
| Consulate General of France in the London | COVID19: Adaption of consular services from 2 June 2020 onwards | https://uk.ambafrance.org/COVID-19-Adaptation-of-consular-services-from-2-June-2020-onwards |
| The French Government | I am currently abroad | https://www.welcometofrance.com/en/je-suis-a-letranger |
| Consulate General of France in the London | Health Pass and Vaccination in the UK | https://uk.ambafrance.org/NHS-COVID-Pass-now-accepted-in-France |
| **Japan - Total Sources: 5** | | |
| **Source** | **Webpage Title** | **Reference URL** |
| Consulate-General of Japan in Sydney | (PDF) For permanent residents who have difficulty re-entering Japan within the valid period of their re-entry permission due to the impact of the novel coronavirus (COVID-19) (From April 16, 2021) | https://www.sydney.au.emb-japan.go.jp/document/english/visa_info/pr-reentry-16apr.pdf |
| Consulate-General of Japan in Sydney | (PDF) To Anyone Entering/Returning to Japan | https://www.sydney.au.emb-japan.go.jp/document/english/visa_info/entering-app-eng-re.pdf |
| Ministry of Foreign Affairs of Japan | (PDF) Supporting Japanese Nationals Overseas | https://www.mofa.go.jp/files/000527159.pdf |
| Ministry of Foreign Affairs of Japan | Program to Strengthen Livelihood and Business Foundations for Japanese Nationals Overseas and People of Japanese Descent (Nikkei) | https://www.mofa.go.jp/ca/cp/page22e_000949.html |
| Ministry of Foreign Affairs of Japan | Border enforcement measures to prevent the spread of novel coronavirus (COVID-19) | https://www.mofa.go.jp/ca/fna/page4e_001053.html |
| **New Zealand - Total Sources: 5** | | |
| **Source** | **Webpage Title** | **Reference URL** |
| New Zealand Government – Foreign Affairs and Trade | COVID-19: Advice for New Zealanders Overseas | https://www.safetravel.govt.nz/news/covid-19-advice-new-zealanders-overseas |
| New Zealand Government | Health and Wellbeing | https://covid19.govt.nz/health-and-wellbeing/ |
| New Zealand Government – Foreign Affairs and Trade | Financial Difficulties | https://www.safetravel.govt.nz/financial-difficulties |
| New Zealand Government | New Zealanders overseas | https://covid19.govt.nz/travel/new-zealanders-overseas/#border-controls-on-arrival |
| New Zealand Government – Foreign Affairs and Trade | COVID-19 and international travel | https://www.safetravel.govt.nz/covid-19-coronavirus |
| **Singapore - Total Sources: 8** | | |
| **Source** | **Webpage Title** | **Reference URL** |
| Ministry of Foreign Affairs Singapore | COVID-19 Travel Restrictions | https://www.mfa.gov.sg/Services/Singapore-Citizens/COVID-19-Travel-Restrictions |
| Singapore Government Agency – Safe Travel | Overview | https://safetravel.ica.gov.sg/sc-pr/overview |
| Singapore Government Agency – Singapore Global Network | About us | https://singaporeglobalnetwork.gov.sg/about-us/ |
| Singapore Government Agency – Singapore Global Network | COVID-19 Situation in Singapore | https://singaporeglobalnetwork.gov.sg/resources/covid-19/ |
| Singapore Government Agency – Safe Travel | Requirements and process | https://safetravel.ica.gov.sg/sc-pr/requirements-and-process |
| Ministry of Foreign Affairs Singapore | General Consular Assistance | https://www.mfa.gov.sg/Services/Singapore-Citizens/I-Need-Help-Overseas/General-Consular-Assistance |
| Ministry of Foreign Affairs Singapore | I Need Help Overseas | https://www.mfa.gov.sg/Services/Singapore-Citizens/I-Need-Help-Overseas. |
| Ministry of Foreign Affairs Singapore | Assistance for Singaporeans | https://www.mfa.gov.sg/Overseas-Mission/Geneva/Consular-Services/Assistance-for-Singaporeans |
| **Spain - Total Sources: 15** | | |
| **Source** | **Webpage Title** | **Reference URL** |
| Government of Spain - The Ministry of Foreign Affairs, European Union and Cooperation (MFA) | Ministry of Foreign Affairs creates platform to facilitate contact between stranded travellers and Spanish residents overseas | https://www.lamoncloa.gob.es/lang/en/gobierno/news/Paginas/2020/202004043aloja-service.aspx |
| MFA | Your Consulate Can Help You: Register | http://www.exteriores.gob.es/Portal/en/ServiciosAlCiudadano/SiEstasEnElExtranjero/Paginas/TuConsuladoPuedeAyudarte.aspx |
| Government of Spain – Spain Travel Health (SpTH) | Prevention measures against COVID-19 | https://www.spth.gob.es/more |
| SpTH | FAQs – About the Spain Travel Health program; Health requirements to enter Spain and prevention measures against COVID-19 | https://www.spth.gob.es/faq?tab=2 |
| MFA | Press Release - Ministry of Foreign Affairs schedules five new flights to help Spaniards trying to return home | http://www.exteriores.gob.es/Portal/en/SalaDePrensa/NotasdePrensa/Paginas/2020_NOTAS_P/20200424_NOTA062.aspx |
| MFA | Press Release - 15 Spanish citizens repatriated from Colombia | http://www.exteriores.gob.es/Portal/en/SalaDePrensa/NotasdePrensa/Paginas/2020_NOTAS_P/20201218_NOTA222.aspx |
| MFA | Press Release - Spain sends humanitarian aid to Nepal to tackle the COVID-19 pandemic | http://www.exteriores.gob.es/Portal/en/SalaDePrensa/NotasdePrensa/Paginas/2021_NOTAS_P/20210519_NOTA109.aspx |
| MFA | Press Release - Ministry of Foreign Affairs to arrange arrival of a dozen flights from Latin America before end of May | http://www.exteriores.gob.es/Portal/en/SalaDePrensa/NotasdePrensa/Paginas/2020_NOTAS_P/20200522_NOTA074.aspx |
| MFA | Press Release - Ministry of Foreign Affairs arranges for more than 200 Spaniards to return from India and Indonesia | http://www.exteriores.gob.es/Portal/en/SalaDePrensa/NotasdePrensa/Paginas/2020_NOTAS_P/20200405_NOTA44.aspx |
| MFA | Press Release - Ministry of Foreign Affairs arranges return of 275 passengers from Philippines | http://www.exteriores.gob.es/Portal/en/SalaDePrensa/NotasdePrensa/Paginas/2020_NOTAS_P/20200423_NOTA61.aspx |
| MFA | What to do in an Emergency | http://www.exteriores.gob.es/Portal/en/ServiciosAlCiudadano/SiViajasAlExtranjero/Paginas/QueDeboHacerEnCasoDeEmergencia.aspx |
| MFA | What a Consulate can/cannot do for you | http://www.exteriores.gob.es/Portal/en/ServiciosAlCiudadano/SiViajasAlExtranjero/Paginas/QuePuedeNoPuedeHacerUnConsuladoPorTi.aspx |
| MFA | Consular Assistance | http://www.exteriores.gob.es/Consulados/SYDNEY/en/ServiciosConsulares/Pages/AsistenciaConsular.aspx |
| MFA | Press Release - Ministry of Foreign Affairs to complete repatriation of Spaniards on close to 50 flights | http://www.exteriores.gob.es/Portal/en/SalaDePrensa/NotasdePrensa/Paginas/2020_NOTAS_P/20200430_NOTA065.aspx |
| MFA | Other Consular services | http://www.exteriores.gob.es/Portal/en/ServiciosAlCiudadano/SiEstasEnElExtranjero/Paginas/OtrosServiciosConsulares.aspx |
| **Thailand - Total Sources: 8** | | |
| **Source** | **Webpage Title** | **Reference URL** |
| Ministry of Foreign Affairs, Kingdom of Thailand (MFA) | Registration process for Thai nationals | https://coethailand.mfa.go.th/regis/step?language=enth |
| MFA | The Protection of Thai Nationals Abroad Division's Main Responsibilities | https://www.mfa.go.th/en/page/protection-of-thai-nationals?menu=5e2022a9c4281a00b65968b3 |
| The Civil Aviation Authority of Thailand | The Notification on Conditions for Aircraft Permission to Enter Thailand | https://www.caat.or.th/en/archives/51895 |
| Ministry of Public Health (MOPH) | (PDF) Managing the new wave of the Covid-19 Epidemic Ministry of Public Health, January 2021 | https://ddc.moph.go.th/viralpneumonia/eng/file/main/en_Thailand%20Covid-19%20plan_MOPH_2021.pdf |
| Royal Thai Consulate-General | e-Visa and Certificate of Entry (COE) arrangement | https://thaiconsulatela.org/en/visa/related-procedures-in-requesting-for-a-certificate-of-entry-and-visa-to-enter-thailand/ |
| MFA | Press release – The Royal Thai Embassy and team in Singapore organized facilitated repatriation flights for Thai nationals returning home | https://www.mfa.go.th/en/content/120260-the-royal-thai-embassy-and-team-thailand-20. in-singapore-organized-and-facilitated-repatriation-flights-for-thai-nationals-returning-home |
| Royal Thai Embassy, Washington D.C | COVID-19 Situation in Thailand | https://thaiembdc.org/covid-19inthailand/ |
| MOPH | (PDF) Thailand's Experience in the COVID-19 Response | https://ddc.moph.go.th/viralpneumonia/eng/file/pub_doc/LDoc9.pdf |
| **UK - Total Sources: 9** | | |
| **Source** | **Webpage Title** | **Reference URL** |
| The Government of the United Kingdom | Waiting to return to the UK during coronavirus (COVID-19) | https://www.gov.uk/guidance/coronavirus-covid-19-staying-where-you-are-if-you-cannot-return-to-the-uk |
| The Government of the United Kingdom | Find a British embassy, high commission or consulate | https://www.gov.uk/world/embassies |
| The Government of the United Kingdom | Healthcare support for when you are waiting to return to the UK during coronavirus (COVID-19) | https://www.gov.uk/guidance/healthcare-support-for-when-you-are-unable-to-return-to-the-uk-during-coronavirus-covid-19 |
| The Government of the United Kingdom | Wellbeing and mental health if you're abroad during the coronavirus (COVID-19) pandemic | https://www.gov.uk/guidance/wellbeing-and-mental-health-during-the-coronavirus-covid-19-pandemic |
| The Government of the United Kingdom | Financial assistance abroad | https://www.gov.uk/government/publications/financial-assistance-abroad/financial-assistance-abroad |
| The Government of the United Kingdom | Travel abroad from England during coronavirus (COVID-19) | https://www.gov.uk/guidance/travel-advice-novel-coronavirus#when-youre-abroad |
| The Government of the United Kingdom | Entering the UK | https://www.gov.uk/uk-border-control |
| British Parliament | What to do if you’re affected by a crisis overseas | https://www.gov.uk/guidance/how-to-deal-with-a-crisis-overseas?step-by-step-nav=8c0c7b83-5e0b-4bed-9121-1c394e2f96f3 |
| The Government of the United Kingdom | Travel abroad: step by step | https://www.gov.uk/travel-abroad |
| **U.S - Total Sources: 13** | | |
| **Source** | **Webpage Title** | **Reference URL** |
| U.S Department of State – The Bureau of Consular Affairs | Smart Traveler Enrollment Program | https://step.state.gov/ |
| The Bureau of Consular Affairs | Emergencies | https://travel.state.gov/content/travel/en/international-travel/emergencies.html |
| The Bureau of Consular Affairs | COVID-19 Frequently Asked Questions For U.S. Citizens | https://travel.state.gov/content/travel/en/international-travel/emergencies/covid-19-faqs-for-us-citizens.html |
| The Bureau of Consular Affairs | Emergency Financial Assistance for U.S. Citizens Abroad | https://travel.state.gov/content/travel/en/international-travel/emergencies/emergency-financial-assistance.html#:~:text=The%20U.S.%20Department%20of%20State's,who%20are%20temporarily%20destitute%20abroad |
| The Bureau of Consular Affairs | What the Department of State Can and Can't Do in a Crisis | https://travel.state.gov/content/travel/en/international-travel/emergencies/what-state-dept-can-cant-do-crisis.html |
| The Bureau of Consular Affairs | Crisis Abroad: Be Ready | https://travel.state.gov/content/travel/en/international-travel/emergencies/what-can-you-do-crisis-abroad.html |
| The Bureau of Consular Affairs | Traveler's Checklist | https://travel.state.gov/content/travel/en/international-travel/before-you-go/travelers-checklist.html#checklist_parentitem_1 |
| The Bureau of Consular Affairs | Information for U.S. Citizens about a U.S. Government-Assisted Evacuation | https://travel.state.gov/content/travel/en/international-travel/emergencies/for-evacuated-citizens.html |
| U.S Embassy & Consulates in Australia | Medical Assistance | https://au.usembassy.gov/u-s-citizen-services/doctors/ |
| U.S Embassy & Consulates in Australia | COVID-19 Information | https://au.usembassy.gov/covid-19-information/ |
| Centers for Disease Control and Prevention | Mental Health and Travel | https://wwwnc.cdc.gov/travel/page/mental-health |
| Centers for Disease Control and Prevention | After International Travel | https://www.cdc.gov/coronavirus/2019-ncov/travelers/after-travel-precautions.html |
| The Bureau of Consular Affairs | Presidential Proclamations on Novel Coronavirus | https://travel.state.gov/content/travel/en/News/visas-news/presidential-proclamation-coronavirus.html |
| Centers for Disease Control and Prevention | International Travel During COVID-19 | https://www.cdc.gov/coronavirus/2019-ncov/travelers/international-travel-during-covid19.html |
