# Supplementary material for: How have governments supported citizens stranded abroad due to COVID-19 travel restrictions? A comparative analysis of the financial and health support in eleven countries
Source: BMC Infect Dis. 2022 Feb 20;22:161. doi: 10.1186/s12879-022-07155-2 (PMC8858437; doi:10.1186/s12879-022-07155-2)
Supplement: Supplementary file 2 — Additional file 2. Comparison of Border control and re-entry information provided to citizens abroad of 11 countries as of June, 2021. [file 12879_2022_7155_MOESM2_ESM.docx]

**Additional file 2.** Comparison of Border control and re-entry information provided to citizens abroad of 11 countries as of June, 2021

| **Country** | **Border Restrictions** | **Who can enter?** | **Quarantine and screening requirements** |
| --- | --- | --- | --- |
| **Australia** | Australia’s borders are closed with international restrictions in place, except for repatriation flights. A cap exists on how many passengers can enter each week into each state. | 1. Australian citizens (and immediate family members) 2. Permanent residents 3. Travellers from New Zealand that have been there at least 14 days prior to departure | - Must present a negative PCR test within 72hours prior to boarding - Mandatory 14-day quarantine and frequent PCR testing at own expense for all arrivals (price varies depending on state) |
| **Canada** | Canada’s borders are closed with international restrictions in place, except for the special circumstances outlined in ‘Who can Enter’ | 1. Canadian citizen 2. Permanent residents of Canada 3. Protected person 4. Foreign nationals who fulfill the following criteria   -immediate or extended family member of a citizen who intends to stay longer than 15days  -Registered on the Canada’s Indian act  -Those travelling for compassionate reasons as approved by the Public Health Agency  -Participation in an International Single-Sport event  -From August 9, U.S citizens and permanent residents can travel to Canada if they are fully vaccinated  -Some temporary foreign workers, international students, people awaiting approval for permanent residency, transiting passengers, and many others if permission is granted and they are except from the travel restrictions | - All air arrivals over the age of 5 must present a negative molecular test within 72hours prior to boarding or for land arrivals a negative molecular test must be presented within 14-90days prior to arrival - Must download ArriveCan app and input all information prior to arriving - Mandatory 3-day hotel quarantine, booked through ArriveCan for all arrivals and frequent PCR testing at own expense for all arrivals |
| **Fiji** | Fiji’s borders are closed with international restrictions in place, except for repatriation flights. | 1. Fijian citizens 2. Permanent residents 3. Other nationals only with grounds for exemption approved by the Office of the Prime Minister | - Must present a negative PCR test within 72hours and emailed to immigration prior to boarding - Mandatory quarantine at own expense for non-Fijian citizens arrivals - Additional PCR test conducted during quarantine |
| **France** | French borders are open with restrictions based on which country travellers enter from on a traffic light system. | 1. Vaccinated travellers from any country on the orange and green list and those accompanying them – must have proof 2. Non-vaccinated travellers from a list of ‘Green countries’ 3. Non-vaccinated travellers from a list of ‘Orange or Red” countries can only enter France if they possess pressing grounds for travel | - Must present a negative PCR test within 72hours prior to boarding   Or   - Present evidence of vaccination status alongside a sworn statement of no symptoms or contact with a COVID-19 confirmed case - On arrival into France, there are no restrictions for fully vaccinated people from green or orange listed countries. 7 days self-isolation for those entering from an orange country, and a possible random test upon arrival and finally a 10-day quarantine for those entering from a red country along with a test upon arrival into France for unvaccinated travellers and a 7day self-isolation for vaccinated travellers |
| **Japan** | Japan’s borders are closed with international restrictions in place, except for repatriation flights. | 1. Japanese citizens 2. Permanent residents, unless they have stayed in the list of high risk countries and will be denied entry. | - Must present a negative PCR test within 72hours prior to boarding - Mandatory 14-day quarantine (designated by the quarantine station chief) with a negative PCR test on arrival if returning from a country with COVID-19 cases - If passengers are arriving from a country with COVID-19 the first 3 days of quarantine will be at a specifically designed facility and can continue quarantine at home after a negative test on day 3. |
| **New Zealand** | New Zealand’s borders are closed with international restrictions in place, except for repatriation flights. | 1. New Zealand citizens 2. Permanent residents  - and immediate family members who must travel with a New Zealand Citizen (partner/spouse, legal guardians and dependent children under 24) and also need approval from Immigration New Zealand | - Must present a negative PCR test within 72hours and emailed to immigration prior to boarding - Mandatory 14-day quarantine at own expense at designated facilities, and must be booked through the Managed Isolation Allocation System and present a voucher of booking prior to boarding |
| **Singapore** | Singapore’s borders have controls and restrictions in place but is open for tourism from certain countries. | 1. Singapore citizens 2. Permanent Residents 3. The following are limited based on travel history and region and must be approved prior to arrival from the relevant government agency (MOE, MOH):   -Long Term Pass Holders  -Immediate relatives of Singapore citizens or residents  -Short term business trips  -Short-term tourism  -Death or illness compassionate visit | - Must present a negative PCR test within 72hours prior to boarding – exemption for this occurs if traveller has been in a low-risk country for 21days prior to departure - -Depending on history of travel prior to arrival, a Stay-At-Home-Notice may be given |
| **Spain** | Spain’s borders are open with restrictions based on which country travellers enter from. | 1. Vaccinated travellers from any region, with proof. 2. Travellers from a country with low incidence – no restrictions 3. All passengers entering Spain from a high-risk region must provide proof of vaccination, a negative PCR test within 72hours prior to departure or proof of COVID-19 recovery. | - All travellers must complete a Health Control Form prior to entering Spain |
| **Thailand** | Thailand’s borders have controls and restrictions in place. They are currently closed for tourism at this time. | 1. Thai citizens 2. Those with exemption given by the Prime minister 3. Those with consular or diplomatic missions 4. Temporary entry of those carrying necessary goods 5. Immediate family of Thai citizens 6. Those with work permits (and family) 7. International students 8. Those in need of medical treatment in Thailand 9. Those who are allowed entry due to an arrangement with the foreign country | - Must present a negative PCR test within 72hours (if needed by the airline) - Mandatory 14-day quarantine at own expense at designated facilities, with 3 PCR tests during stay OR if in possession of a Thai Passport and ticked from Thai Airways can quarantine for free in a premise provided by the State Quarantine authorities - All passengers must apply for a Certificate of Entry prior to departure (COE) |
| **UK** | UK borders are open with restrictions based on which country travellers enter from on a traffic light system. These rules vary between England, Northern Island, Scotland and Wales. | 1. Travellers from all countries on the green and amber list with restrictions 2. For countries on the red list, you can only enter if you have the right to live in the UK (Citizens, permanent residents) | - Depending on the country you have departed from, your vaccination status and where you are entering (England, Northern Island, Scotland or Wales), there are different rules, PCR test and quarantine hotel requirements. - Quarantine will be required for 10days, at own expense if arriving from a country on the red list. |
| **U.S** | America’s borders are closed with international restrictions in place, except for repatriation flights and limited land border restrictions for essential travel. | 1. U.S citizens 2. Permanent residents 3. Other exceptions exist include foreign diplomates, family of U.S citizens and others. | - Must present a negative PCR test within 72hours or a certificate of recovery prior to boarding - Another test is required between 3-5days after arrival for all passengers except for those with a COVID-19 recovery certificate - Passengers must isolate for 7 days after arrival unless you test negative and are fully vaccinated, or you have a COVID-19 recover certificate. |
| PCR, Polymerase Chain Reaction ;MOM, Ministry of Manpower; MOH, Ministry of Health. | | | |
